# Monitoring of cerebral blood flow during hypoxia‐ischemia and resuscitation in the neonatal rat using laser speckle imaging

**DOI:** 10.14814/phy2.12749

**Published:** 2016-04-14

**Authors:** Thomas Wood, Elisa Smit, Elke Maes, Damjan Osredkar, Mari Falck, Maja Elstad, Marianne Thoresen

**Affiliations:** ^1^ Department of PhysiologyInstitute of Basic Medical SciencesUniversity of OsloOsloNorway; ^2^ Neonatal NeuroscienceSchool of Clinical SciencesUniversity of BristolBristolUnited Kingdom

**Keywords:** Cerebral blood flow, hypoxia‐ischemia, neonatal, oxygen, resuscitation

## Abstract

Neonatal hypoxic‐ischemic encephalopathy (HIE) is associated with alterations in cerebral blood flow (CBF) as a result of perinatal asphyxia. The extent to which CBF changes contribute to injury, and whether treatments that ameliorate these changes might be neuroprotective, is still unknown. Higher throughput techniques to monitor CBF changes in rodent models of HIE can help elucidate the underlying pathophysiology. We developed a laser speckle imaging (LSI) technique to continuously monitor CBF in six postnatal‐day 10 (P10) rats simultaneously before, during, and after unilateral hypoxia‐ischemia (HI, ligation of the left carotid artery followed by hypoxia in 8% oxygen). After ligation, CBF to the ligated side fell by 30% compared to the unligated side (*P* < 0.0001). Hypoxia induced a bilateral 55% reduction in CBF, which was partially restored by resuscitation. Compared to resuscitation in air, resuscitation in 100% oxygen increased CBF to the ligated side by 45% (*P* = 0.033). Individual variability in CBF response to hypoxia between animals accounted for up to 24% of the variability in hemispheric area loss to the ligated side. In both P10 and P7 models of unilateral HI, resuscitation in 100% oxygen did not affect hemispheric area loss, or hippocampal CA1 pyramidal neuron counts, after 1‐week survival. Continuous CBF monitoring using LSI in multiple rodents simultaneously can screen potential treatment modalities that affect CBF, and provide insight into the pathophysiology of HI.

## Introduction

Perinatal asphyxia is a major cause of morbidity and mortality, with 1–2 per 1000 infants born at term experiencing perinatal asphyxia that results in hypoxic‐ischemic encephalopathy (HIE) (Kurinczuk et al. 2010). Despite the introduction of therapeutic hypothermia (TH) as the standard of care for infants with HIE, 40–50% of affected infants will still be left with significant neurological disability (Edwards et al. 2010). The etiology of HIE is thought to be at least partly due to the combined effects of reduced oxygen delivery to the brain (hypoxia) and a concurrent fall in cardiac output or cerebral blood flow (ischemia). Indeed, preclinical animal models used to study neonatal HIE often include a combination of hypoxia with either arterial occlusion or systemic hypotension (Rice et al. 1981; Thoresen et al. 1996c; Sabir et al. 2012; Alonso‐Alconada et al. 2015). The hypoxic‐ischemic (HI) insult results in a primary energy failure, characterized by an inability to regenerate high‐energy phosphates, and loss of regulated ATP‐dependent processes in the brain, such as maintenance of ion homeostasis (Hansen 1985; Thoresen et al. 1995; Wassink et al. 2014). Though current treatments, such as TH, focus on the secondary effects of HI‐induced brain injury in asphyxiated neonates, there is ongoing interest in studying cerebral blood flow (CBF) changes during HI and resuscitation, as knowledge regarding the restoration of CBF following neonatal HI is still limited. This includes the effect of resuscitation using supplemental oxygen versus resuscitation in air. Current guidelines recommend starting neonatal resuscitation with 21–30% oxygen and titrating upwards, as long‐term neurological outcomes from human trials using higher oxygen concentrations are lacking, and data from animal models is often inconsistent (Perlman et al. 2015). Resuscitation in 100% oxygen can more rapidly restore CBF after HI (Presti et al. 2006), but is thought to increase oxidative damage in the at‐risk brain (Solberg et al. 2012). Further preclinical investigations into CBF changes during HI and resuscitation are therefore needed to help understand the etiology of HIE, as well as investigate the physiological effects of potential treatments for at‐risk infants during the perinatal and resuscitation period.

The model most commonly used to investigate neonatal HIE is the Vannucci model of unilateral HI in postnatal day seven (P7) to P10 rats and mice (Rice et al. 1981; Sheldon et al. 1998; Sabir et al. 2012; Burnsed et al. 2015; Patel et al. 2015). In this model, the topography of cerebral damage is largely influenced by regional blood flow loss after ligation and during hypoxia (Vannucci et al. 1988). Changes in CBF during HI in this model were initially investigated by Vannucci et al. using iodo[^l4^C]antipyrine in P7 rats, a method that has recently been replicated in P9 mice (Vannucci et al. 1988; Ek et al. 2015). However, this approach involves sacrificing each animal at the point of measurement, which prevents longitudinal data collection. Magnetic resonance imaging (MRI) and laser Doppler flowmetry (LDF) have also been used to monitor blood flow changes during and after HI in the Vannucci model, but can generally only take measurements from one animal at a time, or over short periods of time. This reduces the number of animals that can feasibly be studied, as well as the temporal resolution and ability to track dynamic CBF changes (Qiao et al. 2004; Presti et al. 2006). One method that could potentially overcome these limitations is laser speckle imaging (LSI) (Briers 2001). This method has been used to study CBF changes before and after unilateral HI in P7 rats, and during and after HI in P9 mice (Ohshima et al. 2012; Taniguchi et al. 2014). Though these previous studies only investigated one animal at a time, there is scope to take measurements from multiple animals at a time due to the use of a field of laser light rather than individual probes. Here, we describe for the first time a method utilizing LSI to monitor bilateral changes in CBF in multiple rodents simultaneously before, during, and after unilateral HI. In order to test the feasibility of using this method to explore methods of resuscitation and their effect on CBF, a comparison of CBF changes in rats resuscitated in 21% or 100% oxygen, and how those changes correlate with neuropathology, was also investigated.

## Materials and methods

### Animals

Experiments performed with P7 Wistar rats (Charles River Laboratories, Sulzfeld, Germany) were reviewed and approved by the University of Oslo's animal ethics research committee. Experimental procedures performed with P10 Wistar rats (Charles River Laboratories, Margate, Kent, UK) were carried out under Home Office license in accordance with UK regulations, and approved by the University of Bristol's animal ethical review panel. All experiments were carried out in accordance with the approved protocols as well as the ARRIVE (Animal Research: Reporting in vivo Experiments) guidelines. Prior to treatment, pups of both sexes were randomized across litter, sex, and weight to resuscitation in either 21% oxygen (room air) or 100% oxygen. All pups were kept in an animal facility with their dams under a 12 h:12 h dark:light cycle at 21°C environmental temperature. Dams had access to food and water ad libitum, and pups were checked for health daily.

### Laser speckle contrast analysis

To determine the feasibility of bilateral CBF measurement throughout a unilateral HI insult and subsequent resuscitation in neonatal rats, the Vannucci HI protocol was adapted in order to allow continuous monitoring using laser speckle imaging (LSI). Speckle contrast analysis was first described by Fercher and Briers (Fercher and Briers 1981). Continuous LSI has since been used to monitor CBF in rodents (Dunn et al. 2001; Taniguchi et al. 2014), and has been validated for measuring real‐time (micro)vascular blood flow during surgery (Hecht et al. 2009; McGuire and Howdieshell 2010). Tissue exposed to a laser field will backscatter the light, producing a speckle pattern that is detected by a charged‐couple device (CCD) camera. As the laser light comes into contact with tissue or particles that are in motion, such as red blood cells (RBCs), the speckle pattern becomes blurred, where the degree of blurring is directly related to the speed of movement. In a stationary tissue, the speckle pattern is blurred by the movement of RBCs, and the degree of blurring is determined by the speed and concentration of RBCs. The speckle pattern is then used to provide a map of superficial blood flow velocities in real‐time (Briers 2001). The total amount of flow at any given time point is quantified using the arbitrary units (au) of “flux” (Fercher and Briers 1981). As the skull of the P10 rat is less than 160 *μ*m thick, the near‐infrared laser is able to penetrate around 500 *μ*m into the cerebral tissue (Levchakov et al. 2006; Ohshima et al. 2012). Flux values can then be monitored continuously over predefined regions of interest across the tissue.

**Figure 1:**
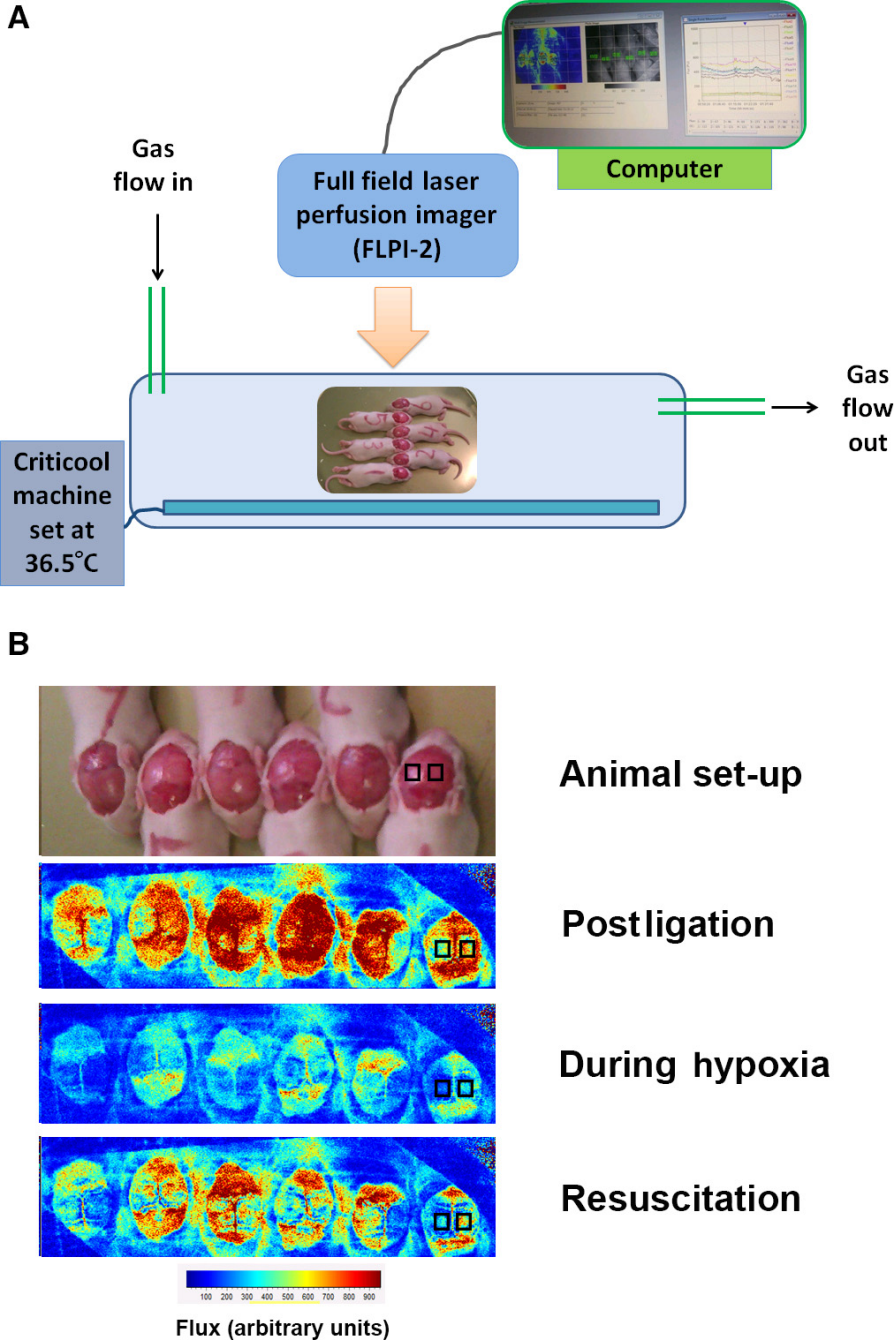
Laser speckle imaging. Experimental set‐up (A) and representative images (B) using full‐field LSI to determine hemispheric blood flow (measured in arbitrary units of “flux”). The lower panel shows six animals with scalps exposed, within the hypoxia chamber. Animals faced in alternating directions to minimize breathing restrictions. Cerebral blood flux measurements (1) after ligation; (2) during hypoxia; and (3) after 10 min of resuscitation in 21% oxygen are shown. Representative bilateral regions of interest over the ligated (L) and unligated (R) hemispheres are shown in the animal on the right.

For LSI measurements, 43 rat pups underwent unilateral HI at P10 (Rice et al. 1981). Ligation of the left carotid artery was performed under anesthesia with 3% isoflurane in a 2:1 gas mixture of NO_2_/O_2,_ supplied via a nose cone, as previously described (Osredkar et al. 2015). After carotid artery ligation and 30 min recovery with the dam, pups received an intraperitoneal (i.p) injection of ketamine (40 mg/kg; Vetalar‐V, Pfizer Animal Health Division, Tadworth, UK) and medetomidine hydrochloride (0.5 mg/kg; Medetor, Virbac Animal Health, Bury St. Edmund's, UK). Isoflurane completely abolishes autoregulation of CBF, but etomidate or ketamine/xylazine anesthesia partially preserves CBF autoregulation (Wang et al. 2010). The dose was chosen to allow the animals to self‐ventilate, but still provide adequate anesthesia during the whole experimental period, as any movement would interfere with LSI readings. Once fully anaesthetized, a midline incision was made across the scalp, and the scalp removed. In groups of six, pups were placed on a metal tray within the hypoxia chamber, on top of a servo‐controlled water‐filled mat (CritiCool, MTRE, Yavne, Israel), with one animal carrying a rectal temperature probe (IT‐21, Physitemp Instruments, Clifton, NJ). The chamber was sealed, and ventilated with 21% oxygen until the pups reached the target rectal temperature of 36.5°C. During this time, a laser speckle contrast imager (FLPI‐2, Moor Instruments, Axminster, UK) was placed over the clear polycarbonate lid of the chamber with the exposed scalps within the field of view of the camera (Fig. 1A). Flux measurement software (Moor FLPI v1.1) was provided by the manufacturer, which calculates flux relative to incident ambient light. This allows flux measurements to be compared across the whole laser field regardless of position relative to the camera, and prevents error caused by curvature of the target tissue. Black plastic shields were placed around the camera and chamber to prevent any direct light entering or reflecting from the surface of the chamber and interfering with flux measurements. One virtual region of interest (ROI) was placed over each cerebral hemisphere (12 total), posterior to the bregma, and either side of the sagittal suture (Fig. 1B). Continuous flux values were recorded every 4 sec, and then averaged for each minute of the experiment.

Baseline flux measurements from each region of interest (one over either hemisphere in each animal) were taken for 15 min before the chamber was flushed with 8% oxygen to initiate the 27 min period of hypoxia. During hypoxia, core temperatures were continuously recorded, and rectal temperature was maintained at ±0.2°C of the target temperature. Due to the combined cardiorespiratory effects of anesthesia plus hypoxia, pilot experiments showed a significant increase in intra‐hypoxic mortality if hypoxia was continued for more than 30 min (data not shown). At the end of the hypoxia period, the pups were resuscitated by flushing the chambers with either 21% or 100% oxygen for 30 min followed by a further 45 min recovery in 21% oxygen. Pups were then removed from the chamber and sacrificed.

### Neuropathology after sham laser speckle imaging

A second group of P10 animals (*n* = 44) underwent unilateral HI using the model modified for laser speckle contrast analysis. Animals were sedated using ketamine‐medetomidine anesthesia, exposed to 27 min of hypoxia (8% oxygen), and resuscitated in either air or 100% oxygen as described above. The scalp was not removed, and no flux measurements were taken. At the end of the resuscitation period, medetomidine anesthesia was reversed using atipamezole hydrochloride (Antisedan 5 mg/mL, Pfizer Animal Health Division, Tadworth, UK). Pups were returned to the dam, and sacrificed after 1 week to determine hemispheric area loss.

### Area loss analysis

One week after HI, rats were sacrificed via transcardiac perfusion with saline followed by 10% neutral‐buffered formalin under isoflurane/N_2_O anesthesia. Brains were harvested and kept in 10% neutral‐buffered formalin for 5 days until further processing. Coronal 3 mm blocks were cut through the brain using a standard rat brain matrix (ASI Instruments Inc., Warren, MI), and embedded in paraffin. Sections of 5 *μ*m were stained with hematoxylin and eosin (H&E). Two sections from each of the two neighboring blocks best representing cortex, hippocampus, basal ganglia and thalamus, were scanned (Epson Perfection V750 Pro), and virtual slides were exported as 600 dpi images. To measure the area of brain tissue loss, the optical density and hemispheric area of each section was analyzed with ImageJ software (ImageJ, version 1.46r, National Institutes of Health, Bethesda, MD) by an individual who was blinded to group allocation. The average percentage of area loss was calculated from the two neighboring sections by using the following formula: [1 − (area left/area right)] × 100. Percent hemispheric area loss has previously been shown to be highly correlated with a formal neuropathology score in this model (Sabir et al. 2012).

### Effect of resuscitation in 100% oxygen after unilateral HI in P10 rats

To examine the effect of resuscitation in 100% oxygen or 21% oxygen on hemispheric area loss without anesthesia during hypoxia, unilateral HI injury was induced in P10 pups (*n* = 37) using a modified Vannucci protocol (Rice et al. 1981). Pups underwent ligation of the left carotid artery as described above, before being returned to the dams for at least 30 min, and then exposed to 75 min of hypoxia with 8% oxygen at 36.5°C. This model produces a “moderate” injury equivalent to our previously published P7 model, with around 30–40% left hemispheric loss (Sabir et al. 2012). Immediately after the end of hypoxia, the chamber was flushed with either 21% or 100% oxygen depending on the preassigned randomization. During the recovery period, target rectal temperature was increased to 37°C, as previously described (Bona et al. 1998; Vannucci et al. 1999). After 30 min, the group receiving 100% oxygen was switched to 21% oxygen, and both groups remained in the chambers for an additional 5 h before being returned to the dams (Thoresen et al. 1996b; Sabir et al. 2012). After 1 week of survival, the pups were sacrificed, and hemispheric area loss was determined as described above.

### Effect of resuscitation in 100% oxygen after unilateral HI in P7 rats

To compare potential differences in effect of resuscitation in air or 100% oxygen between the P10 and P7 model, the experimental protocol was repeated in a separate group of animals at P7 (*n* = 94). Postnatal day seven (P7) Wistar rat pups have been traditionally used in the Vannucci model of unilateral HI, and are considered to have an equivalent level of brain maturation to the 34–36‐week‐old human neonate, with P10 rats thought to be closer to those born at full term (40–42 weeks) (Tucker et al. 2009; Semple et al. 2013). Animals underwent ligation and hypoxia as described above, with a hypoxia time of 100 min at a target temperature of 36°C in order to provide a “moderate” injury (Sabir et al. 2012). After resuscitation in either 21% or 100%, rats were maintained at 37°C in air for 5 h before being returned to their dams for 1 week of survival and analysis of area loss.

### Immunohistochemistry

A subset of brains from the P7 experiments was processed for immunohistochemistry. Paraffin‐embedded tissue was deparaffinized in xylene and rehydrated in decreasing concentrations of ethanol. Antigen retrieval was performed in citrate buffer (pH 6.0), using a PT link instrument (Dako, Denmark). After blocking with 10% goat serum, a primary mouse antibody against NeuN (1:500; Millipore, MA) was applied overnight at room temperature. Slices were rinsed with PBS and incubated for 1 h at room temperature with secondary Alexa Fluor 568 (Invitrogen, 1:500) antibodies. Slides were rinsed again, and coverslipped with ProLong Gold with 4′,6‐diamidino‐2‐phenylindole (DAPI, Invitrogen). Sections were scanned with a virtual microscopy scanner (Axio Scan.Z1, Carl Zeiss, Jena, Germany) in fluorescence mode with a plan apochromatic 20× lens. Virtual slides were then exported as high‐resolution tiff images. Three consecutive nonoverlapping ROIs were assessed from the CA1 region of both hippocampi (Osredkar et al. 2015). Cells located within the pyramidal layer, which were clearly within the plane of focus, and positive for both NeuN (neuronal marker) and DAPI (cell nucleus), were counted as neurons. The total number of viable pyramidal neurons across the three ROIs from each hippocampus was summed. Cell counting was performed by an observer blinded to the treatment group. A subset of regions (25%) was reassessed by a second blinded observer, and interobserver reliability was determined to ensure reproducibility.

### Statistical analysis

Pups carrying rectal and skin temperature probes were excluded from the final analysis, as the stress of carrying probes has previously been shown to have a neuroprotective effect (Thoresen et al. 1996a). Area loss and hippocampal neuron counts were compared between the two resuscitation methods using a two‐tailed Mann–Whitney *U*‐test. For preresuscitation (before and during hypoxia) flux measurements, the unligated and ligated sides of all animals were compared in a pairwise manner using the Wilcoxon matched‐pairs signed rank test at 15 min intervals. After the end of hypoxia, the median blood flux value for each animal during both the resuscitation and subsequent recovery period was found. Median flux values from both the ligated and unligated side were then compared between groups using a two‐tailed Mann–Whitney *U*‐test. Statistical analyses were performed using SPSS software version 22 (SPSS Inc., Chicago, IL). Flux measurements are presented as median with range. For area loss measurements, a Hodges–Lehmann median with 95% confidence interval (CI) was calculated (StatXact version 10; Cytel, Cambridge, MA), as previously described (Dalen et al. 2012). To assess the degree to which variability in CBF to the ligated hemisphere during hypoxia might contribute to the overall variability in injury in the model, the coefficient of variation (mean/standard deviation) was calculated for the percent reduction in flux in each animal during hypoxia (prehypoxia flux versus end‐hypoxia flux on the ligated side), and compared to the coefficient of variation for area loss in sham LSI measurement animals resuscitated in air. A *P*‐value <0.05 was considered statistically significant.

## Results

### Resuscitation in 100% oxygen increases flux in the ligated hemisphere

**Figure 2:**
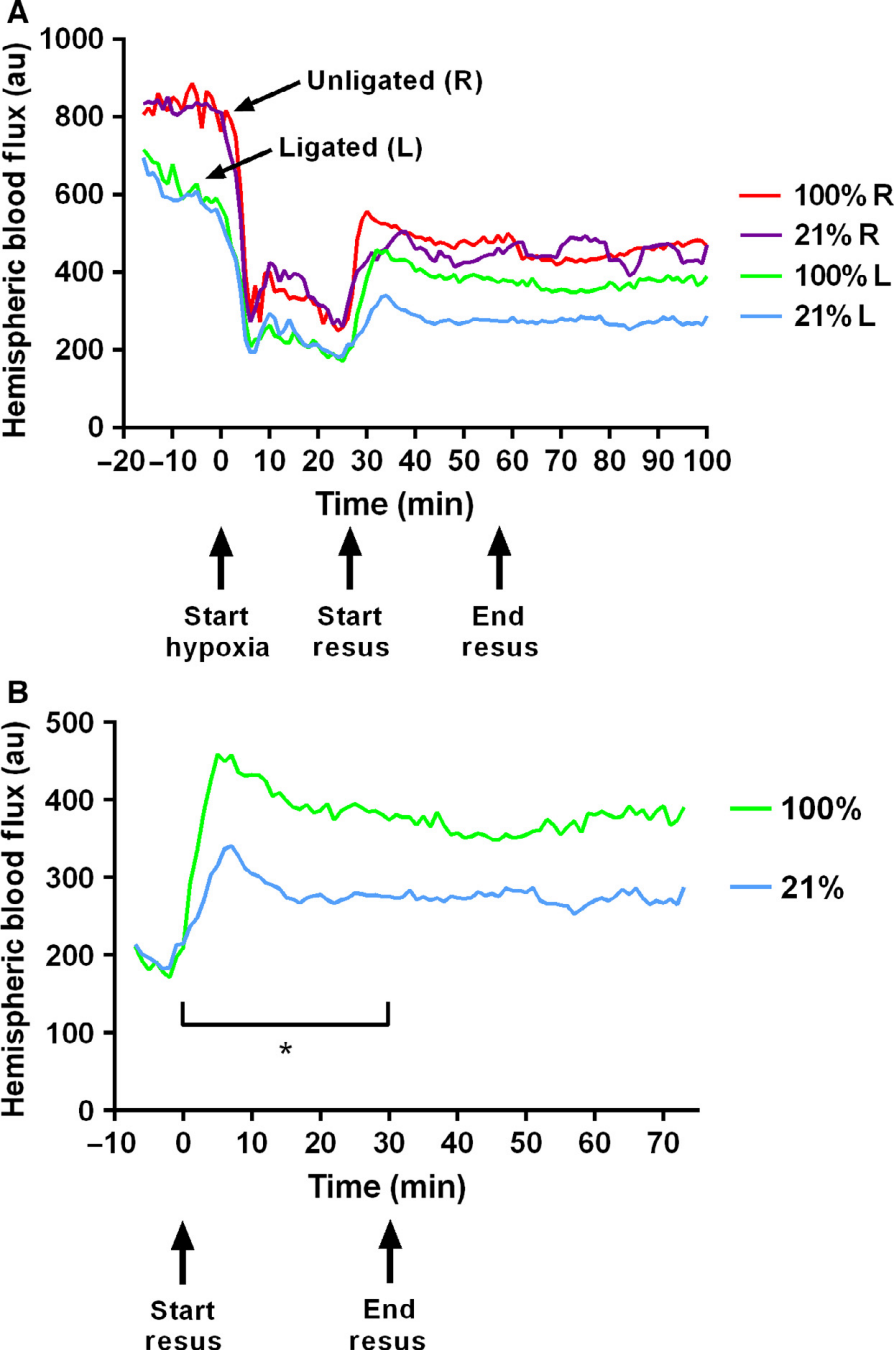
Temporal measurement of hemispheric blood flow. (A) Peripheral cortical blood flow (measured in arbitrary units of “flux”) after ligation, during hypoxia, and during and after resuscitation. Data shows the median flux value every 60 sec on the ligated (L, blue and green lines) and unligated (R, purple and red lines) side in the groups resuscitated in 21% oxygen (*n* = 16) and 100% oxygen (*n* = 15). Arrows indicate the start of hypoxia (27 min at 8% oxygen), start of resuscitation, and end of resuscitation (both groups switched to 21% oxygen). The ligated hemispheres displayed significantly lower flux measurements than the unligated hemispheres (*P* < 0.0001). (B) Median flux value every 60 sec on the ligated side in the two groups immediately before, during, and after resuscitation. During resuscitation, the 100% oxygen group (green line) had significantly greater flux measurements compared to the group resuscitated in 21% oxygen (blue line). There was a continued trend towards greater flux in the 100% oxygen group during the subsequent recovery period (*P* = 0.093). *Denotes significant difference between the two groups (*P* = 0.033).

During the flux measurement protocol, one pup died during ligation and 11 died during hypoxia (26% mortality), leaving 31 pups resuscitated with either 21% (*n* = 16) or 100% (*n* = 15) oxygen. In order to examine the effect of ligation and hypoxia on flux, both groups were analyzed together until the end of hypoxia. Flux on the ligated side was significantly lower (*P* < 0.0001) than the unligated side throughout (Fig. 2A). Immediately after ligation, median (range) flux measurement was 689 au (458–117, *n* = 31) on the ligated side, and 830 au (505–1229, *n* = 31) on the unligated side (Table 1). Hypoxia resulted in a bilateral 55% decrease in flux (*P* < 0.0001), which was not completely reversed by resuscitation. After the end of hypoxia, median flux measurements were taken for each animal during the resuscitation and recovery periods, and the two treatment groups compared (Table 2). During early resuscitation, both groups showed a degree of bilateral reactive hyperemia. On the ligated (left) side during the resuscitation period, median flux in the group resuscitated in 100% oxygen (396 au, range 260–592 au) was 45% greater than in the group resuscitated in 21% oxygen (274 au, 228–540 au). The difference between the two groups was statistically significant (*P* = 0.033, Fig. 2B). During the recovery period, a trend (*P* = 0.093) toward increased flux in the group resuscitated in 100% oxygen remained. There was no difference in flux on the unligated side between the two treatment groups. Flux to the unligated side remained similar in both groups during both the resuscitation and recovery periods, which lasted 30 min and 45 min, respectively.

**Table 1:** Effect of ligation and hypoxia on cerebral blood flux. Median (with range) flux values for the ligated and unligated hemisphere before and during hypoxia. Pairwise comparisons between the ligated and unligated hemisphere within each animal were performed

Timepoint	Cerebral blood flux (au)	
	Ligated	Unligated
After ligation (−15 mins)	689 (458–1166)	830 (505–1229)*
Prehypoxia (0 mins)	571 (337–1004)	813 (491–1108)*
Early hypoxia (+5 mins)	258 (115–717)†	367 (130–750)*, †
Mid‐hypoxia (+15 mins)	251 (115–530)	381 (193–863)*
End hypoxia	189 (91–311)	272 (76–466)*

*Denotes *P* < 0.0001 compared to ligated side.

^†^Denotes *P* < 0.0001 compared to prehypoxia value.

**Table 2:** Effect of resuscitation gas on hemispheric flux. Median (with range) flux values for the ligated and unligated hemisphere during resuscitation with 21% oxygen or 100% oxygen, and subsequent recovery. Median values for each animal, over both time periods, were compared between groups

Period	Cerebral blood flux (au)			
	21% Oxygen		100% Oxygen	
	Ligated	Unligated	Ligated	Unligated
Resuscitation	274 (228–539)	442 (327–657)	396 (260–592)*	501 (342–736)
Recovery	278 (182–462)	478 (290–651)	368 (236–520)	441 (328–679)

*Denotes *P* < 0.05 compared to ligated hemisphere in the 21% oxygen group.

### Resuscitation in 100% oxygen does not influence area loss after sham flux measurements

**Figure 3:**
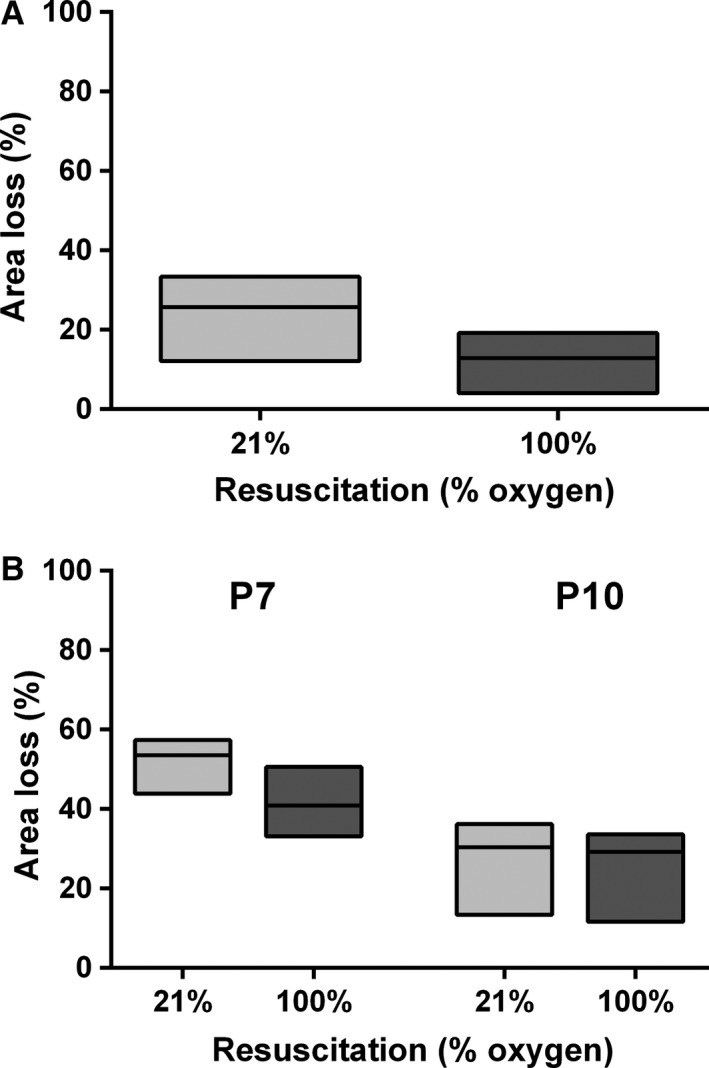
Hemispheric area loss after resuscitation in 21% or 100% oxygen. (A) Area loss after control flux measurements. Box plot (Hodges–Lehmann median with 95% CI) of hemispheric area loss in P10 rats after unilateral HI under anesthesia, and sham CBF measurements, before resuscitation in either 21% oxygen (*n* = 11) or 100% oxygen (*n* = 13). (B) Area loss after hypoxia‐ischemia in P7 and P10 rats. Box plot (Hodges–Lehmann median with 95% CI) of hemispheric area loss in P7 and P10 rats after unilateral HI and resuscitation in either 21% oxygen or 100% oxygen.

As animals that underwent flux measurements had been instrumented and could not survive for neuropathology, a sham group (*n* = 44) underwent anesthesia and HI with the same protocol to assess the effect of this model on neuropathology. Nineteen pups died during hypoxia, and one pup randomized to 21% oxygen died during the survival period. Median area loss was 25.7% (12.1–33.4%, *n* = 11) in the 21% oxygen group, and 12.9% (4.0–19.2%, *n* = 13) in the 100% oxygen group (Fig. 3A). However, the difference in area loss between the two groups was not significant (*P* = 0.12).

### Resuscitation with 100% oxygen after HI does not affect area loss in P10 rat pups

Due to the potential confounding effects of anesthesia in the context of HI, resuscitation in 21% and 100% oxygen were compared in a standard Vannucci model in P10 rat pups (*n* = 37). Four pups were excluded because they carried a temperature probe, four died during hypoxia, and one pup in each group died during the follow‐up period. Area loss (Hodges–Lehmann median with 95% confidence interval) was 30.4% (13.4–36.2%, *n* = 14) in the 21% oxygen group, and 29.2% (11.6–33.6%, *n* = 13) in the 100% oxygen group (Fig. 3B). No difference in area loss was seen between the two different treatment groups (*P* = 0.59).

### Resuscitation with 100% oxygen after HI does not affect area loss or mortality in P7 rat pups

We have previously shown that resuscitation in 100% oxygen in the P7 Vannucci model resulted in greater neuropathological injury compared to resuscitation in 21% oxygen (Dalen et al. 2012). Therefore, the lack of difference between the two groups in the P10 model was unexpected. As a result of this, we repeated the experimental protocol in the P7 model. Similar to the P10 model, initial results showed that resuscitation in 100% oxygen (*n* = 21) after HI did not affect mortality or hemispheric area loss compared to resuscitation in 21% oxygen (*n* = 22) in the P7 model. The protocol was repeated in the P7 model to ensure that the contradictory results were likely to be accurate. No effect of resuscitation in 100% oxygen was seen in the second experimental run, so the results from both experiments were combined for the final analysis (Fig. 3B). A total of 98 rat pups were randomized to receive either 21% oxygen (*n* = 49) or 100% oxygen (*n* = 49). Four pups died during ligation, and eight were excluded as they carried temperature probes. No animals died during or after resuscitation (0% mortality). Area loss was 53.5% (43.9–57.4%, *n* = 42) and 40.9% (33.1–50.6%, *n* = 44) in the groups resuscitated in 21% oxygen and 100% oxygen, respectively, which was not significantly different (*P* = 0.16).

### Resuscitation with 100% oxygen does not affect CA1 hippocampal neuron survival in P7 rat pups

**Figure 4:**
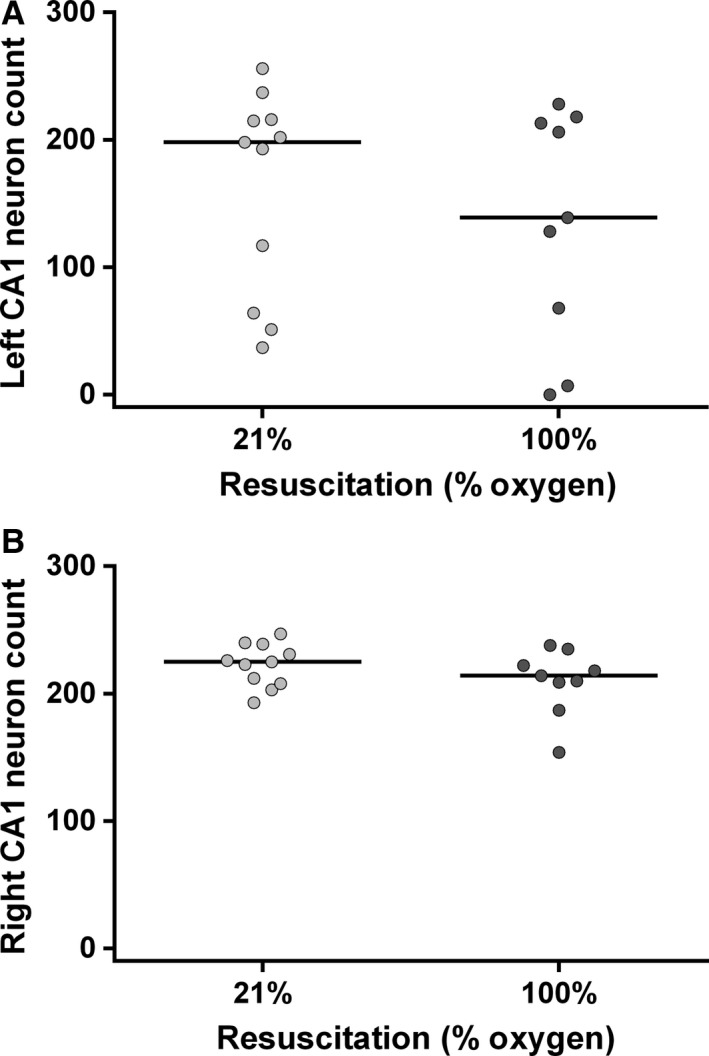
Hippocampal CA1 neuron counts after unilateral HI. Ipsilateral (A) and contralateral (B) hippocampal CA1 pyramidal neuron counts after resuscitation in either 21% oxygen (*n* = 11) or 100% oxygen (*n* = 9).

**Figure 5:**
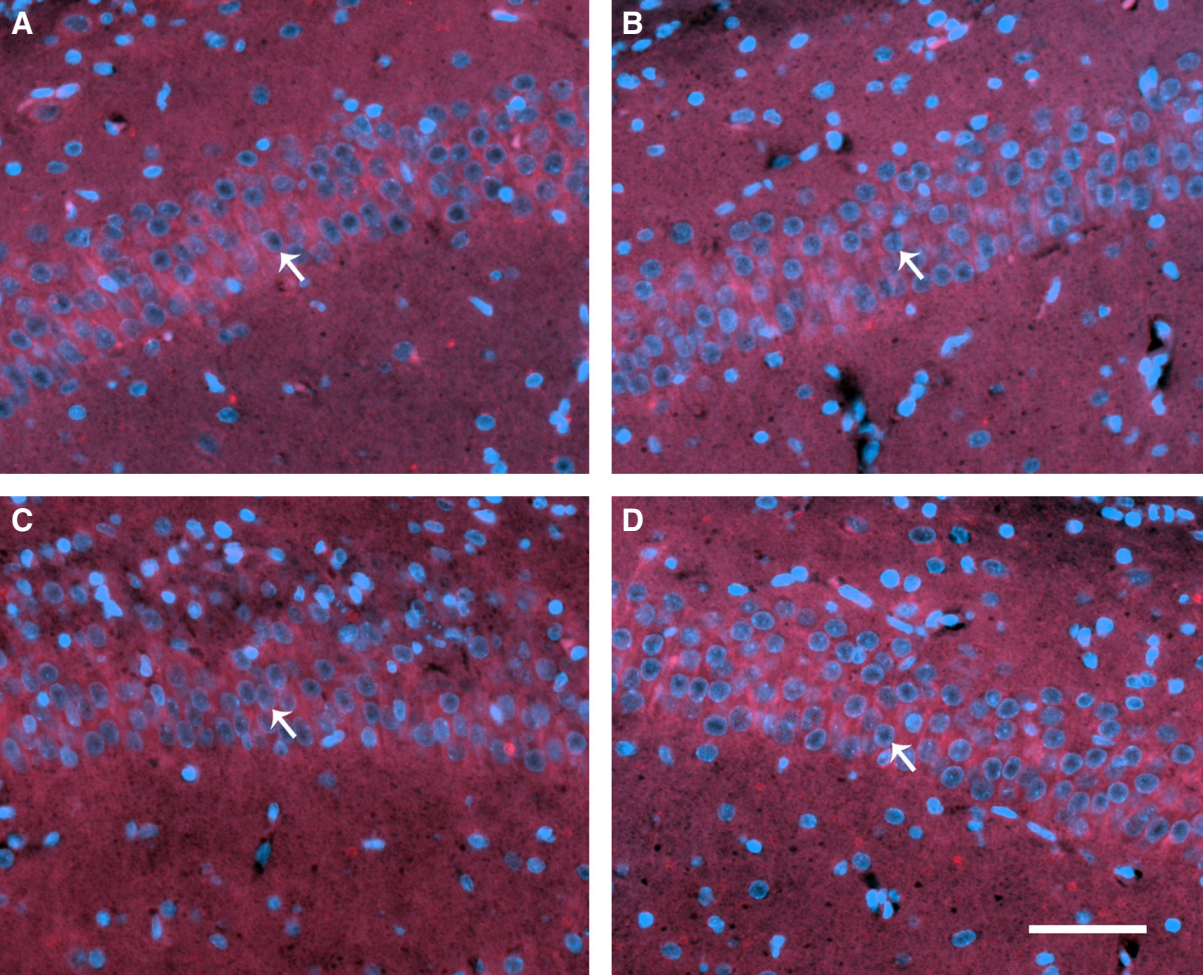
Hippocampal immunohistochemistry. Representative CA1 hippocampal regions of interest from each hemisphere in the 21% oxygen group (A, B), and 100% oxygen group (C, D). The ligated hemisphere is shown on the left (A, C), with the unligated hemisphere on the right (B, D). Arrows show typical viable pyramidal neurons with large, round nuclei (DAPI, blue), and NeuN costaining (red). Scale bar (bottom right) represents 50 *μ*m.

To investigate whether resuscitation in 100% oxygen increases the susceptibility of vulnerable hippocampal pyramidal neurons to the effects of HI in P7 rats, immunohistochemistry and counting of viable CA1 pyramidal neurons was performed (Fig. 4A). Across three consecutive ROIs, median (range) total neuronal cell count on the ligated side was 198 (37–256, *n* = 11) in the 21% oxygen group, and 139 (0–228, *n* = 9) in the 100% oxygen group. No difference in ipsilateral hippocampal neuronal cell counts was seen between the two groups (*P* = 0.62). In the right hemisphere (unligated side), median total cell count was 225 (193–247, *n* = 11) in the 21% oxygen group, and 214 (154–238, *n* = 9) in the 100% oxygen group (Fig. 4B). No difference in neuron count was found between the two groups. Representative hippocampal regions of interest are shown in Figure 5.

### Cerebral blood flux variability in response to hypoxia

For each animal, the percent reduction in flux to the ligated side at the end of hypoxia (compared to the final prehypoxia value) was calculated. Mean (standard deviation) flux reduction to the ligated side was 64.8% (7.7%), giving a coefficient of variation of 0.156 (15.6%). In sham flux measurement animals resuscitated in air, the coefficient of variation for area loss after 1‐week survival was 64.0%, with a ratio between the two of 0.24.

## Discussion

Asphyxiated infants often experience a combination of ischemia, including changes in CBF, and systemic hypoxia, which leaves them at high risk for long‐term neurodevelopmental disability despite active treatment (Jacobs et al. 2013; Wassink et al. 2014). As the Vannucci rodent model of neonatal HI has previously helped provide robust evidence for the use of treatments such as TH in asphyxiated neonates, it is a good candidate for investigating CBF changes in the setting of HI, as well as screening the effect of other potential clinical treatments. Here, we demonstrate for the first time a method of monitoring peripheral CBF (measured as flux) simultaneously in multiple animals, using LSI during unilateral HI in P10 rat pups. We show that ligation causes a reduction in flux to the ipsilateral side, with subsequent hypoxia inducing a bilateral fall in flux. Flux values were partially restored by resuscitation, with a greater effect of resuscitation in 100% oxygen, compared to resuscitation in 21% oxygen, on the ligated side. When the same resuscitation protocols were investigated outside the context of LSI measurements, resuscitation in 100% oxygen did not affect short‐term pathology in either our P10 or P7 Vannucci rat model of HI.

A number of groups have investigated cerebrovascular changes in preclinical models of HIE. This is a complex area, where CBF changes in HI will depend on both the species being examined, as well as the experimental protocol. The results of studies investigating the effect of HI on CBF in rodent models are diverse, and a summary of those discussed here is provided in Table 3. After unilateral ligation in our P10 model using LSI, flux in the ligated hemisphere was significantly lower than in the unligated hemisphere, and continued to fall until the start of hypoxia. Hypoxia resulted in a 55% bilateral reduction in flux, which was not fully reversed by resuscitation. These initial results are in relative agreement with early work by Vannucci et al. using radiolabeled iodo[^14^C]antipyrine, which showed that unilateral ligation leads to increased flow in the contralateral hemisphere relative to the ipsilateral hemisphere, with subsequent hypoxia then eliciting a significant drop in ipsilateral blood flow (Vannucci et al. 1988). However, unlike the results presented here, hypoxia in that study did not result in a reduction in perfusion of the contralateral hemisphere below baseline.

**Table 3:** Summary of studies examining cerebral blood flow variables in the Vannucci model. Summary of referenced studies including blood flow measurement technique, effect of ligation and hypoxia, and resuscitation in air or 100% oxygen

Study (Technique)	Species (Age)	Anesthesia	Effect on cerebral blood flow							
			hHypoxia		During Hypoxia		Resuscitation (air)		100% Oxygen	
			Ligated side	Unligated side	Ligated side	Unligated side	Ligated side	Unligated side	Ligated side	Unligated side
Vannucci et al. (1988)	Rat	N/A	↔	↑ (Frontal Cortex)	↓ (Global)	↔	N/A		N/A	
iodo[14C]antipyrine	(P7)				↑ (Cerebellum)	↑ (Cerebellum)				
Ek et al. (2015)	Mouse	N/A	N/A		↓	↑	↓ (2 h)	↓ (2 h)	N/A	
iodo[14C]antipyrine	(P7)						↔ (6 h)	↔ (6 h)		
Presti et al. (2006)	Mouse	N/A	N/A		↓	↔	↔	↔	↑	↔
LDF	(P7/P8)									
Qiao et al. (2004)	Rat	Isoflurane	↓ (Global)	↔	↓	↔	↔	↔	N/A	
MRI	(P7)									
Ohshima et al. (2012)	Rat	Isoflurane	↓ (Global)	↔	N/A		↑	↔	N/A	
LSI	(P7)									
Taniguchi et al. (2014)	Mouse	Isoflurane	↓	N/A	↓	↓	↓	↓	N/A	
LSI	(P9)							↑ (vs hypoxia)		
Buckley et al. (2015)	Rat	N/A	↓	↓	N/A		↑ (immediate)	↑ (immediate)	N/A	
DCS	(P10)						↔ (2 h)	↔ (2 h)		
This study	Rat	Ketamine +	↓	↔	↓	↓	↓	↓	↑ (vs air)	↔ (vs air)
LSI	(P10)	Medetomidine					↑ (vs hypoxia)	↑ (vs hypoxia)		

LDF, laser Doppler flowmetry; MRI, magnetic resonance imaging; LSI, laser speckle imaging; DSC, diffuse correlation spectroscopy.

Blood flow changes are relative to preligation baseline values unless otherwise stated. ↑ = increased, ↓ = decreased, ↔ = no change, N/A = not applicable (not measured).

One main issue with using an injectable radioisotope in this manner is that the animal must be sacrificed in order to make the CBF measurement. As the Vannucci model is known to produce highly variable results (Patel et al. 2015), a technique that allows for longitudinal measurements and can be used to detect dynamic changes in CBF is desirable. In P7 rats, magnetic resonance imaging (MRI), showed a significant reduction in cortical blood flow during HI only on the ipsilateral side, which normalized slowly over 24 h of recovery (Qiao et al. 2004). In P7/P8 mice, LDF found a minimal effect of HI on blood flow to the unligated hemisphere, but a hypoxia‐induced decrease in flow to the ligated side, which was reversed within 10 min of resuscitation in air (Presti et al. 2006). Taniguchi et al. used LSI in the P9 mouse during hypoxia. They describe a bilateral reduction in blood flow during hypoxia as we do, which was partially restored by reoxygenation (Taniguchi et al. 2014). Taken together, findings in rodent models of HI suggest that unilateral ligation results in a reduction of CBF to the ligated hemisphere relative to the unligated hemisphere, with a further decrease caused by subsequent hypoxia. However, depending on the exact model and measurement technique used, there is enough heterogeneity in the results to make it difficult to generalize the effects of unilateral HI on CBF.

A major confounding variable during the measurement of blood flow during HI is the use of anesthesia. Most investigators have used isoflurane during measurements in rodents (Qiao et al. 2004; Ohshima et al. 2012; Taniguchi et al. 2014). We chose ketamine/medetomidine as LSI studies have shown that isoflurane completely abolishes autoregulation of CBF, but etomidate or ketamine/xylazine partially preserve it (Wang et al. 2010). Anesthesia may also reduce the depth and frequency of respiration, and alter CBF by increasing pCO_2_ levels. By comparison, LDF does not require anesthesia other than for initial insertion of the probes. However, it is not known how the stress of being awake with probes in situ will affect blood flow, and we have previously shown that stress affects the results of the Vannucci model (Thoresen et al. 1996a). Another interesting method that does not require anesthesia is diffuse correlation spectroscopy (DCS), a near‐infrared multiple light‐scattering technique using a handheld probe that can penetrate deeper than LSI, though over a smaller total surface area (Buckley et al. 2015). Buckley et al. recently used DCS to assess CBF after unilateral HI, showing reoxygenation caused a bilateral reactive hyperemia, similar to what we describe. This suggests that, though an effect of the anesthesia cannot be excluded, it is likely that some degree of cerebral autoregulation remained in our animals.

Another issue that must be taken into account when discussing serial CBF measurements is temporal resolution. A single MRI scanner taking measurements from multiple animals is time intensive, which reduces the number of possible measurement time points. In this regard, continuous light‐based techniques such as LDF, DCS, and LSI have a large advantage. Compared to LDF and DCS, our set‐up can measure blood flow changes in six rats simultaneously. This technique can also be used during hypoxia, which is not currently the case with DCS. With our LSI equipment, we are able to take flux measurements as frequently as every 0.1 sec, which allows for very sensitive resolution and rapid tracking of CBF changes.

Using this method, we were able to investigate the degree to which variability in CBF responses to hypoxia between animals may account for the high degree of variability seen in the Vannucci model. Comparing end‐hypoxia flux values to immediate prehypoxia flux values within each animal allowed us to determine individual decreases in CBF to the ligated hemisphere in response to hypoxia. The coefficient of variation for this CBF response across all animals was 15.6%, compared to 64% for area loss measurements within the sham P10 group that underwent anesthesia and HI followed by 1‐week survival (ratio of 0.24). This suggests that, within that particular protocol at least, individual variability in CBF response to HI may account for up to 24% of the variability in pathology seen in the model. Though we were not able to measure preligation CBF values, both hypoxia and unilateral ligation are required to generate the injury seen in the Vannucci model (Rice et al. 1981), and the combined effect of the two is still captured within our data. Vannucci et al. previously showed that regional variability of ischemia in response to HI correlated with the relative susceptibility of each region to neuropathological damage in this model (Vannucci et al. 1988), but with higher throughput methods such as simultaneous LSI, we may be able to increasingly understand the effect of individual CBF responses, and how those influence final pathology.

We also investigated the potential of LSI to compare treatments that may affect CBF by resuscitating rats in either 21% or 100% oxygen after unilateral HI. After the end of hypoxia and during the resuscitation period, animals resuscitated in 100% oxygen had a greater increase in ipsilateral hemispheric blood flow, a trend which persisted into the recovery period. One of the potential benefits of resuscitation in 100% oxygen in this model may therefore be more rapid recovery of CBF after HI, which may or may not be beneficial depending on both anatomy and degree of injury or circulatory collapse (Matsiukevich et al. 2010). As is likely to be the case in humans, part of the variability of the Vannucci model may be due to different changes in CBF between animals. This was evident in the high degree of variability in flux measurements across both treatment groups. As methods that monitor CBF usually rely on relative, rather than absolute changes in CBF during HI, a method such as LSI that allows for monitoring of CBF in multiple animals at once can provide more reliable interpretation of the effects of different treatment strategies.

Despite the differences in flux changes in the two resuscitation groups, no difference was seen in terms of hemispheric area loss in the P10 models. Hemispheric area loss and hippocampal CA1 pyramidal counts in the P7 model were also not different between the two treatment groups. This latter result is in opposition to previous work done by our group, where immediate resuscitation in 100% oxygen was seen to increase hippocampal injury (though not injury to the cerebral cortex or basal ganglia) (Dalen et al. 2012). In this study, much larger group sizes were used to minimize the effect of variability in the model, which potentially increases the reliability of the current data. Other studies have also shown conflicting results with resuscitation in 100% oxygen after unilateral HI including no difference between groups (Bagenholm et al. 1996), or benefit from resuscitation in 100% oxygen compared to 21% oxygen (Presti et al. 2006; Calvert and Zhang 2007). This indicates that the relationship between method of resuscitation and outcome after HI is likely to be complex, potentially depending on exact experimental protocols, strain and species of animal used, and the outcome measures examined. The overall poor quality and contradictory nature of the preclinical data, and lack of robust human data, concerning the effect of cardiopulmonary resuscitation in 100% oxygen has been highlighted in the most recent International Consensus on Cardiopulmonary Resuscitation and Emergency Cardiovascular Care Science (Perlman et al. 2015). We were unable to add any definitive answers in this regard, but the use of 100% oxygen during resuscitation did allow us to look at our ability to detect CBF changes in multiple animals simultaneously, which was our primary goal. Though no overt negative short‐term side effects of resuscitation in 100% oxygen were found in this study, a number of preclinical studies have shown that administration of 100% oxygen after HI brain damage can lead to greater oxidative damage, inflammation, markers of lipid peroxidation, and inappropriate release of mitochondrial metabolites (Solberg et al. 2010, 2012; Perez‐Polo et al. 2011). However, the long‐term effects of these changes are largely unknown, as complex large animals studies often involve very short survival periods, and data from human neonates is lacking (Munkeby et al. 2004). A reliance on short‐term surrogate markers of injury might therefore preclude a long‐term benefit that is not examined in most preclinical studies (Presti et al. 2006). This may be analogous to exercise, where a short‐term increase in oxidative stress improves oxidative and metabolic parameters in a hormetic manner (Gomez‐Cabrera et al. 2008), with administration of antioxidants preventing these long‐term beneficial adaptations (Ristow et al. 2009). This is one important reason why long‐term follow‐up from the original clinical trials comparing resuscitation in 100% oxygen versus room air in asphyxiated neonates is still eagerly awaited (Tan et al. 2005).

This study has some limitations. As with any study where part of the scalp or skull has to be removed to study the brain, it is possible that retracting the scalp increased convection away from the cortex and affected peripheral cortical temperature. With our current setup we were unable to directly measure cortical temperature within the chamber. However, cerebral temperature in the Vannucci model has previously been shown to be within 0.1°C of rectal temperature (Thoresen et al. 1996b), and rectal temperature was closely monitored using the servo‐controlled heating mat directly under the pups. The relative size of the skull at P10 (brain weight around 0.8 g) is also very small compared to the heating capacity of the Criticool mat within the chamber (Kishimoto et al. 1965). Additionally, the design of the chamber was such that the gas input and output ports were as far away from the pups as possible in order to minimize direct gas flow over the skull. Though there may have been an effect on skull surface temperature due to removal of the scalp, all animals in the CBF experiments were exposed to the same conditions, and it is unlikely that the effect of removing the scalp will have had a significant effect on the results.

With regards to the mechanism behind HI‐induced decreases in hemispheric CBF, we have been unable to distinguish between changes due to effects on cerebral vascular resistance versus changes caused by reduced arterial blood pressure. As well as different individual responses to carotid ligation, variability in the cardiac effect of prolonged hypoxia is likely to influence the variability in CBF. For instance, the extent to which resuscitation with 100% oxygen improves return of spontaneous circulation (ROSC) in the neonatal mouse after cardiac arrest is based on the degree of circulatory collapse (Matsiukevich et al. 2010). A more rapid reversal of the cardiosuppressive effect of anesthesia plus hypoxia in this model may well be part of the reason why resuscitation in 100% oxygen provided a greater and more rapid restoration in CBF. This benefit may then offset or override the negative effects of increased reactive oxygen species (ROS) in the 100% oxygen group, explaining the overall lack of difference between the treatment groups. Differences in the experimental protocols that affect degree and pattern of ischemia, and the relative contributions of cerebral vascular resistance and arterial blood pressure to CBF changes, may therefore partly determine whether 100% oxygen is found to be beneficial or not. Using our current LSI setup, animals were unable to survive past the end of the recording period as the scalps had been removed. In the future, adapting the technique to allow the scalp to be sutured back for long‐term assessment of neuropathology would allow better correlation between direct individual changes in blood flow (such as degree of hypoperfusion during hypoxia or subsequent reactive hyperemia) and outcome. Clinical translation of LSI for measurement of CBF in neonates is unlikely to be possible, as it does not penetrate deep enough to measure through the human skull. However, systems regularly used in neonates, such as near‐infrared spectroscopy (NIRS) are too bulky to be used on a neonatal rat. When examining treatments that alter CBF, working with a unilateral HI model is also unlikely to be ideal. Though rats do have a fully developed circle of Willis (Brown 1966), permanent carotid artery ligation does not necessarily reflect the clinical scenario, where the vascular supply remains intact.

In summary, we describe the first imaging method that allows real‐time monitoring of peripheral CBF across the entire brain during and after unilateral HI in multiple rodents simultaneously. Resuscitation in 100% oxygen increases blood flow to the ligated hemisphere, and variability in this response in different experimental protocols may underlie some of the different outcomes seen in this model. Resuscitation in 100% oxygen did not affect short‐term neuropathology, and may assist in recanalization of collapsed vasculature. Future application of this technique will allow for better understanding of the pathogenesis of HI brain injury, as well as identification of certain subsets of infants that may benefit from therapies that more directly affect cerebral blood flow.

## Conflict of Interest

None declared.
